# Resistance to oxidative stress by inner membrane protein ElaB is regulated by OxyR and RpoS

**DOI:** 10.1111/1751-7915.13369

**Published:** 2019-01-17

**Authors:** Yunxue Guo, Yangmei Li, Waner Zhan, Thomas K. Wood, Xiaoxue Wang

**Affiliations:** ^1^ CAS Key Laboratory of Tropical Marine Bio‐resources and Ecology Guangdong Key Laboratory of Marine Materia Medica RNAM Center for Marine Microbiology South China Sea Institute of Oceanology Chinese Academy of Sciences Guangzhou 510301 China; ^2^ University of Chinese Academy of Sciences Beijing 100049 China; ^3^ Department of Chemical Engineering Pennsylvania State University, University Park PA 16802‐4400 USA; ^4^ Department of Biochemistry and Molecular Biology Pennsylvania State University University Park PA 16802‐4400 USA

## Abstract

C‐tail anchored inner membrane proteins are a family of proteins that contain a C‐terminal transmembrane domain but lack an N‐terminal signal sequence for membrane targeting. They are widespread in eukaryotes and prokaryotes and play critical roles in membrane traffic, apoptosis and protein translocation in eukaryotes. Recently, we identified and characterized in *Escherichia coli* a new C‐tail anchored inner membrane, ElaB, which is regulated by the stationary phase sigma factor RpoS. ElaB is important for resistance to oxidative stress but the exact mechanism is unclear. Here, we show that ElaB functions as part of the adaptive oxidative stress response by maintaining membrane integrity. Production of ElaB is induced by oxidative stress at the transcriptional level. Moreover, *elaB* expression is also regulated by the key regulator OxyR via an OxyR binding site in the promoter of *elaB*. OxyR induces the expression of *elaB* in the exponential growth phase, while excess OxyR reduces *elaB* expression in an RpoS‐dependent way in the stationary phase. In addition, deletion of *elaB* reduced fitness compared to wild‐type cells after prolonged incubation. Therefore, we determined how ElaB is regulated under oxidative stress: RpoS and OxyR coordinately control the expression of inner membrane protein ElaB.

## Introduction

Oxidative stress results from an imbalance between respiration and the ability of a biological system to readily detoxify the reactive intermediates and repair the resulting damage to lipids, proteins, RNA, DNA and cell membranes (Farr and Kogoma, 1991; Storz and Imlay, 1999). The effects of oxidative stress may be enhanced in ageing and illness (e.g. cancer, diabetes) (Finkel and Holbrook, 2000; Maritim *et al*., 2003; Halliwell, 2007). Virtually all organisms, including animals, plants and microbes, have complex, evolved defence and repair mechanisms for coping with oxidative stress by activating co‐regulated groups of genes; these defences are conserved through evolution as shown by the use of similar enzymes by both bacteria and eukaryotes, such as Class II AP endonucleases (Demple and Harrison, 1994). *Escherichia coli* has a complex set of responses to H_2_O_2_ since 140 genes are induced by H_2_O_2_, including *dps*,* katG* and *ahpC* (Zheng *et al*., 2001a,b). Therefore, determining the deleterious effects of oxidative stress in bacteria and their cellular defence mechanisms might guide investigations in higher systems.

Previously described mechanisms that allow bacteria to cope with oxidative stress can be divided into two groups. The first group includes those enzymes that remove active oxygen species (e.g. catalases, peroxidases and superoxide dismutases). Catalases and NADH peroxidase (Ahp) play important roles in removing active oxygen species based on phenotypic analysis and direct measurement of H_2_O_2_ clearance (Mishra and Imlay, 2012). Catalases decompose H_2_O_2_ to nontoxic oxygen and water, while Ahp inactivates H_2_O_2_ by reducing it to water with the help of NADH which is converted into the unstable intermediated NAD^+^ (Dolin, 1977; Loewen *et al*., 1985). The second group includes those enzymes that repair damaged cellular components (such as DNA repair enzymes and membrane repair enzymes). A non‐specific DNA‐binding protein, Dps, is highly abundant in the stationary phase for *E. coli*, and it protects cells against oxidative stress by preventing DNA damage *in vivo* and *in vitro* (Martinez and Kolter, 1997), which indicates that certain proteins in *E. coli* play major roles in antioxidant defence during non‐growth stages (Demple, 1991).

Oxidative disruption of membrane integrity is a general phenomenon (Farr *et al*., 1988), and the oxidative stress‐inducible membrane repair response exists in *E. coli*. Ahp (encoded by *ahpCF*), which is dependent on polyamines and protects against H_2_O_2_‐induced stress during entry into the stationary phase (Jung and Kim, 2003), plays an important role in inducible membrane repair by reducing fatty acid hydroperoxides (Farr and Kogoma, 1991). Although the chemistry of lipid peroxidation is well‐established, how oxidative stress‐induced membrane damage alters membrane function is not clear. Some studies have measured the uptake of labelled metabolites by *E. coil* strains after treatment with H_2_O_2_ (Farr *et al*., 1988). In addition, a rapid loss of both proton motive force (ΔP)‐dependent and proton motive force (ΔP)‐independent transport (e.g. twin‐arginine translocation system) is observed within five minutes after cells are treated with 5 mM H_2_O_2_ (Farr *et al*., 1988). However, transport recovers rapidly if the cells are pretreated with 35 μM of hydrogen peroxide, although cells with mutations in *oxyR* and *katG* have no such adaptation, which shows that increased expression of H_2_O_2_ scavenging activities is required to protect cells from membrane damage by oxidative stress (Farr *et al*., 1988). Only a few membrane‐associated proteins have been demonstrated to alter resistance to oxidative stress induced by H_2_O_2_. Inactivation of NADH dehydrogenase, an inner membrane‐bound respiratory protein, increased cell sensitivity to H_2_O_2_ (Storz *et al*., 1990; Farr and Kogoma, 1991). Additionally, RNA polymerase sigma factor RpoH and superoxide dismutase protect the cell from H_2_O_2_ (Carlioz and Touati, 1986; Kogoma and Yura, 1992). Other membrane proteins (e.g. glutathione reductase, porins) are involved in the defence against oxidative stress, but whether they result in cell sensitivity to H_2_O_2_ has not been determined (Farr and Kogoma, 1991). H_2_O_2_ enters cells from the environment, where it can be generated both by the chemical processes and by the deliberate actions of competing organisms (Mishra and Imlay, 2012). For acute toxicity of H_2_O_2_, bacteria use the above defence mechanisms to keep their intracellular concentrations at nanomolar levels (Mishra and Imlay, 2012). In *E. coli*, the permeability of membranes for H_2_O_2_ is substantial (Seaver and Imlay, 2001). Under specific conditions such as in the stationary phase and in the presence of external H_2_O_2_, membranes of certain bacteria show very poor permeability to H_2_O_2_, and these differences can be explained by changes in membrane lipid composition or by diffusion‐facilitating channel proteins or a combination of both (Bienert *et al*., 2006).

C‐tail anchored inner membrane proteins represent a family of poorly studied membrane proteins and play critical roles in membrane traffic, apoptosis and protein translocation (Kalbfleisch *et al*., 2007; Kriechbaumer *et al*., 2009; Pedrazzini, 2009). Recently, we discovered that disruption of a member of this family of proteins, ElaB, reduces stress resistance including resistance to oxidative stress and heat shock, and inactivation of ElaB can also lead to deleterious effects, such as increased persistence in *E. coli* (Guo *et al*., 2017). ElaB is under the direct control of RpoS which is important for the general stress response and several genes encoding enzymes that remove active oxygen species are also regulated by RpoS. However, since ElaB lacks an enzymatic domain, how it protects cells during oxidative stress remains unclear.

In this paper, we designed experiments to uncover the mechanism by which ElaB protects cells against oxidative stress. We demonstrate that ElaB transcription and translation are induced in response to oxidative stress, that the expression of *elaB* is regulated by both OxyR and RpoS by binding of both regulators to the promoter region of *elaB*, and that the regulation of *elaB* by OxyR is RpoS‐dependent. In addition, deletion of *elaB* reduces fitness, and ElaB protects cells against oxidative stress by maintaining membrane integrity.

## Results

### ElaB is induced by oxidative stress

**Figure 1:**
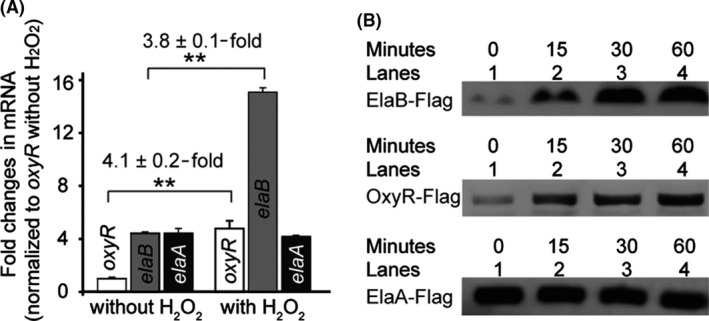
Expression of ElaB is induced by oxidative stress. A. Overnight cultures of BW25113 wild type (WT) were diluted to a turbidity of 0.05 at 600 nm and cultured at 37°C to a turbidity of 1.0; then, 10 mM H_2_O_2_ was added for 10 min. The expression levels of *elaB*,* oxyR* and *elaA* were quantified, and fold changes were calculated. All the fold changes in genes were normalized to *oxyR* in cells without H_2_O_2_ treatment. For statistical analysis, *P *<* *0.01 is shown in **. B. ElaB was fused with 2× Flag before the stop codon, and cells were cultured and treated with 5 mM H_2_O_2_ at the indicated time points. The expression levels of ElaB‐Flag and OxyR‐Flag were determined with Western blotting with the same amount of total protein (upper and middle panel). The expression levels of ElaA‐Flag under the same conditions were used as a negative control (lower panel).

To uncover the underlying mechanism of how ElaB participates in oxidative stress, we tested the expression of *elaB*, by examining both mRNA and protein levels, in response to oxidative stress. Transcription of *elaB* was upregulated 3.8 ± 0.1‐fold in wild‐type cells treated with 10 mM H_2_O_2_ for 10 min (Fig. 1A). As a positive control, the gene for the oxidative stress regulator, OxyR, was upregulated by 4.1 ± 0.2‐fold (Fig. 1A). As a negative control, expression of *elaA*, directly upstream of *elaB,* was not affected by oxidative stress (Fig. 1A). Furthermore, the expression of *rpoS*, which encodes the RpoS sigma factor that binds to the *elaB* promoter to regulate its expression (Guo *et al*., 2017), was also not significantly changed under these stress conditions (data not shown).

To test the changes at the protein level, we fused a 2× Flag tag at the C‐terminus of ElaB and produced it from the wild‐type chromosome. To determine whether ElaB is functional in the ElaB‐Flag fusion protein, we also made the same fusion in plasmid pMD19‐*elaB‐flag* and found ElaB complements the oxidative stress sensitivity of the *elaB* mutant strain (Fig. S1). Then, a Flag‐specific antibody was used to determine the effects of oxidative stress on ElaB levels. As expected, the fused ElaB‐2× Flag protein in the chromosome was induced significantly when treated with 5 mM H_2_O_2_ for 60 min (Fig. 1B upper panel). As a positive control, OxyR‐2× Flag was also induced under the same condition (Fig. 1B middle panel). As a negative control, we also fused the 2× Flag to the carboxyl terminus of ElaA and found that ElaA levels were not affected (Fig. 1B lower panel). Therefore, *elaB* is upregulated during oxidative stress.

### ElaB maintains cell membrane integrity during oxidative stress

**Figure 2:**
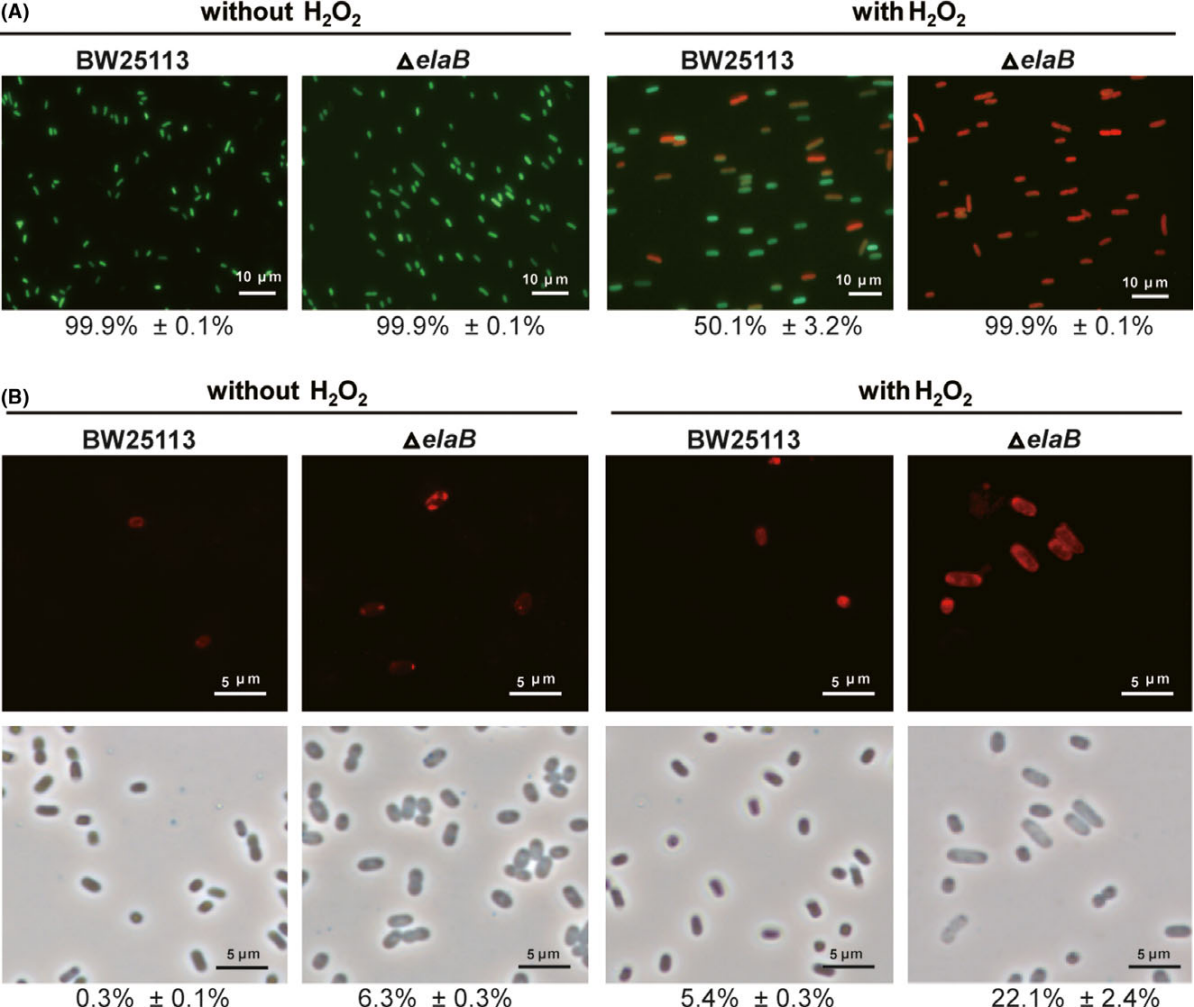
ElaB mutation reduces cell membrane integrity during oxidative stress. BW25113 wild‐type (WT) cells were cultured in the same condition as shown in Fig. 1A. A. Live/Dead staining was performed (live cells appear green, and dead cells appear red/yellow), and the percentages of dead cells were calculated. Cells that were not treated with H_2_O_2_ were used as controls. B. The cells were stained with lipid peroxidation‐specific dye C11‐BODIPY. The upper panels indicate lipid oxidation in the cell membrane, and the lower panels indicate bright‐field views of corresponding upper panels. Percentages of cells with lipid peroxidation were calculated. In A and B, 1000 cells in each culture were observed, and only one representative image for each strain is shown.

Since ElaB is a C‐tail anchored inner membrane, we wanted to explore whether ElaB affects cell membrane integrity during oxidative stress. We utilized the Live/Dead staining kit that uses SYTO 9 and propidium iodide to differentiate between cells with intact membranes (green) and cells with damaged membranes (red and yellow). As expected, the percentage of dead cells was higher in the Δ*elaB* strain (99.5% ± 0.3%) compared to the wild‐type strain (50.1% ± 3.2%) when treated with 10 mM H_2_O_2_ for 10 min (Fig. 2A). As a negative control, both the wild‐type and the Δ*elaB* cells had no dead cells in the absence of H_2_O_2_ treatment (Fig. 2A). In addition, we also stained the membrane of the wild‐type and the Δ*elaB* cells with the plasma membrane‐specific dye red‐fluorescent FM^®^ 4‐64. The plasma membranes of both strains appeared intact and clear in the absence of H_2_O_2_; however, the plasma membrane appeared more diffuse in the presence of H_2_O_2_ for the Δ*elaB* cells (Fig. S2). This suggests that the loss of ElaB affects the cell membrane integrity during oxidative stress.

Lipids are major targets of free radicals generated during oxidative stress, and a primary effect of lipid peroxidation is a decrease in membrane fluidity, which alters membrane properties (Hong *et al*., 2017). We thus tested lipid peroxidation in wild‐type and Δ*elaB* cells during oxidative stress using the fluorescent radio‐probe C11‐BODIPY. In the absence of H_2_O_2,_ 0.3% ± 0.1% of the wild‐type cells showed weak lipid peroxidation while 6.3% ± 0.3% of the Δ*elaB* cells showed lipid peroxidation (Fig. 2B). Furthermore, the lipid peroxidation in the Δ*elaB* cells mainly occurred near or at the cell poles which is the localization site of ElaB (Guo *et al*., 2017). In addition, 22.1% ± 2.4% of the Δ*elaB* cells showed lipid peroxidation in the presence of H_2_O_2_, and it also mainly occurred near or at the cell poles, while 5.4% ± 0.3% of wild‐type cells showed weak lipid peroxidation. Collectively, these microscopic observations demonstrate that the loss of ElaB reduces cell membrane integrity, especially during oxidative stress.

### elaB is regulated by OxyR in a RpoS‐dependent manner

**Figure 3:**
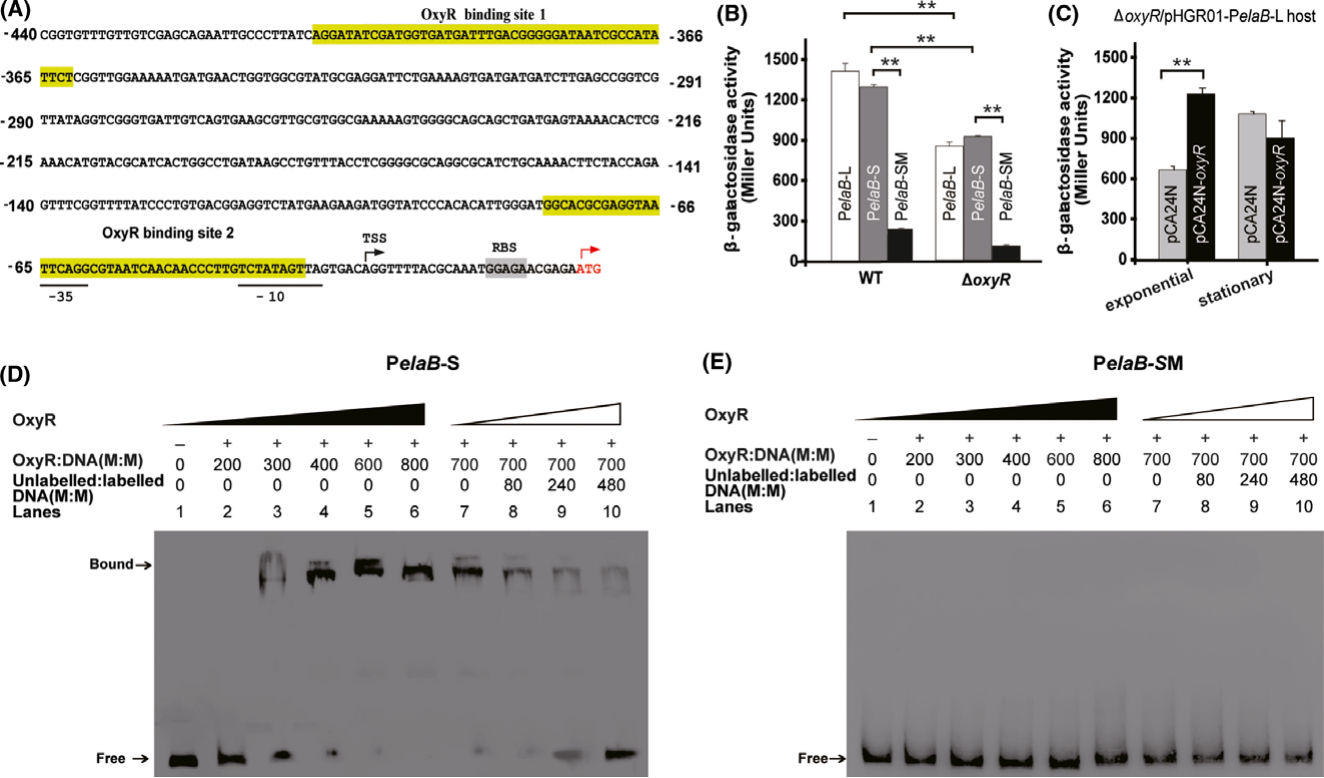
ElaB is regulated by OxyR in *E. coli*. A. The promoter region of *elaB* and the sequences of the probe containing the putative OxyR binding sites are shown. The numbers indicate the locations relative to the start codon A of *elaB*. The predicted binding sites of OxyR are marked. The −10 and −35 regions are highlighted in green and light blue. The transcriptional start site (TSS) is marked with an arrow. The ribosome binding site (RBS) is also highlighted in grey. The start codon of *elaB* is shown in red letters. For the promoter activity assay, the open reading frame (ORF) of *elaB* was replaced by *lacZ* ORF. B. WT and Δ*oxyR* harbouring pHGR01‐P*elaB*‐L (containing OxyR binding sites 1 and 2), pHGR01‐P*elaB*‐S (only containing OxyR binding site 2) and pHGR01‐P*elaB*‐SM (mutation of OxyR binding site 2 in pHGR01‐P*elaB*‐S) cells in the exponential growth phase were collected, and β‐galactosidase activities were evaluated. C. Complementation of *oxyR* via pCA24N‐*oxyR* restored the promoter activity of *elaB* during the exponential growth phase rather than during the stationary phase. For statistical analysis, *P *<* *0.01 is marked as **. D. OxyR binds to the DNA probe (P*elaB*‐S) containing the binding site 2 in a concentration‐dependent manner (lanes 1–6). The addition of unlabelled probe reduced the binding of OxyR to the labelled probe in a concentration‐dependent manner (lanes 7–10). E. OxyR was unable to bind to the mutant DNA probe (P*elaB*‐SM) under the same conditions.

The above results indicated that *rpoS* was not induced during the oxidative stress conditions tested. However, *oxyR* is induced under the same conditions and it is a DNA‐binding transcriptional regulator that controls the expression of antioxidant genes (Zheng *et al*., 2001a,b; Teramoto *et al*., 2013). Thus, we hypothesized that OxyR should be the inducer of *elaB* during oxidative stress. To explore this hypothesis, we first searched for binding sites of OxyR in the 5′ UTR region of *elaB* using the Virtual Footprint (Münch *et al*., 2005) and FGENESB (Softberry, http://www.softberry.com) programs, and two OxyR binding sites were identified (Fig. 3A). We then determined the transcriptional start site (TSS) of *elaB* using 5′ RACE and found that the TSS of *elaB* is located 26 bp upstream of the start codon. To determine whether OxyR regulates the promoter activity, we fused the promoter of *elaB* with different lengths to *lacZ* in the pHGR01 plasmid; the constructed pHGR01‐P*elaB*‐L contains both of the predicted OxyR binding sites (binding site 1 and 2) while pHGR01‐P*elaB*‐S contains only the predicted binding site (binding site 2) near the start codon of *elaB*. We found the promoter activity of pHGR01‐P*elaB*‐L and pHGR01‐P*elaB*‐S in Δ*oxyR* was significantly lower than that in the wild‐type cells (Fig. 3B). Unexpectedly, BW25113 harbouring pHGR01‐P*elaB*‐L and pHGR01‐P*elaB*‐S showed similar promoter activity, and a similar trend was also observed in the Δ*oxyR* host, suggesting that putative binding site 1 should be not important for OxyR regulation of the *elaB* promoter. In addition, binding site 1 is far (about 400 bp) from the start codon of *elaB*, which may be too far away from the *elaB* promoter to exert control. Therefore, we concluded that binding site 2 should be responsible for the regulation of the *elaB* promoter by OxyR. We further mutated sequences in the *elaB* promoter region required for OxyR binding (from 5′ GGCACGCGAGGTAATTCAGGCGTAATCAACAACCCTTG 3′ to 5′ TCTTGAGAGTAAACTTCA GGTCGGACTGTGTGTGTCCA 3′) without altering the −10 and −35 regions to construct pHGR01‐P*elaB*‐SM in order to investigate if the region is important for regulation by OxyR. As expected, the promoter activity of pHGR01‐P*elaB‐*SM in wild‐type cells decreased significantly (Fig. 3B). Unexpectedly, the Δ*oxyR* cells showed the same trend (Fig. 3B). This implied that other regulators may also control the mutated region in the promoter of *elaB*, and RpoS should be one of them (Guo *et al*., 2017). Next, we complemented the *oxyR* mutation in the Δ*oxyR*/pHGR01‐P*elaB*‐L reporter strain by pCA24N‐*oxyR* and tested the promoter activity in the exponential growth and stationary phases. As shown in Fig. 3C, overproducing OxyR via pCA24N‐*oxyR* induced the promoter activity from 660 ± 26 MU for cells with pCA24N to 1220 ± 40 MU for cells with pCA24N‐*oxyR* during exponential growth. However, during the stationary phase, the promoter activity of *elaB* in cells producing OxyR is 900 ± 130 MU, higher than the exponential growth phase. There was no significant difference observed compared to cells harbouring pCA24N. These results indicate that OxyR should regulate the promoter activity of *elaB* in the exponential growth phase.

Since OxyR regulates gene expression by binding to the promoter region, we conducted EMSA using a DNA probe amplified from the promoter of *elaB* (P*elaB‐*S) containing the putative OxyR binding site 2, and using purified OxyR. As shown in Fig. 3D, OxyR bound and shifted the DNA fragment in a dose‐dependent manner (lanes 1–6), and the binding was reduced by the addition of unlabelled probe (lanes 7–10). As a negative control, the same mutant in *elaB* promoter (P*elaB‐*SM) as above for promoter activity assay was not bound and shifted by OxyR (Fig. 3E). Taken together, the transcription and EMSA results indicate, OxyR regulates *elaB* expression by binding to the promoter region of *elaB*.

**Figure 4:**
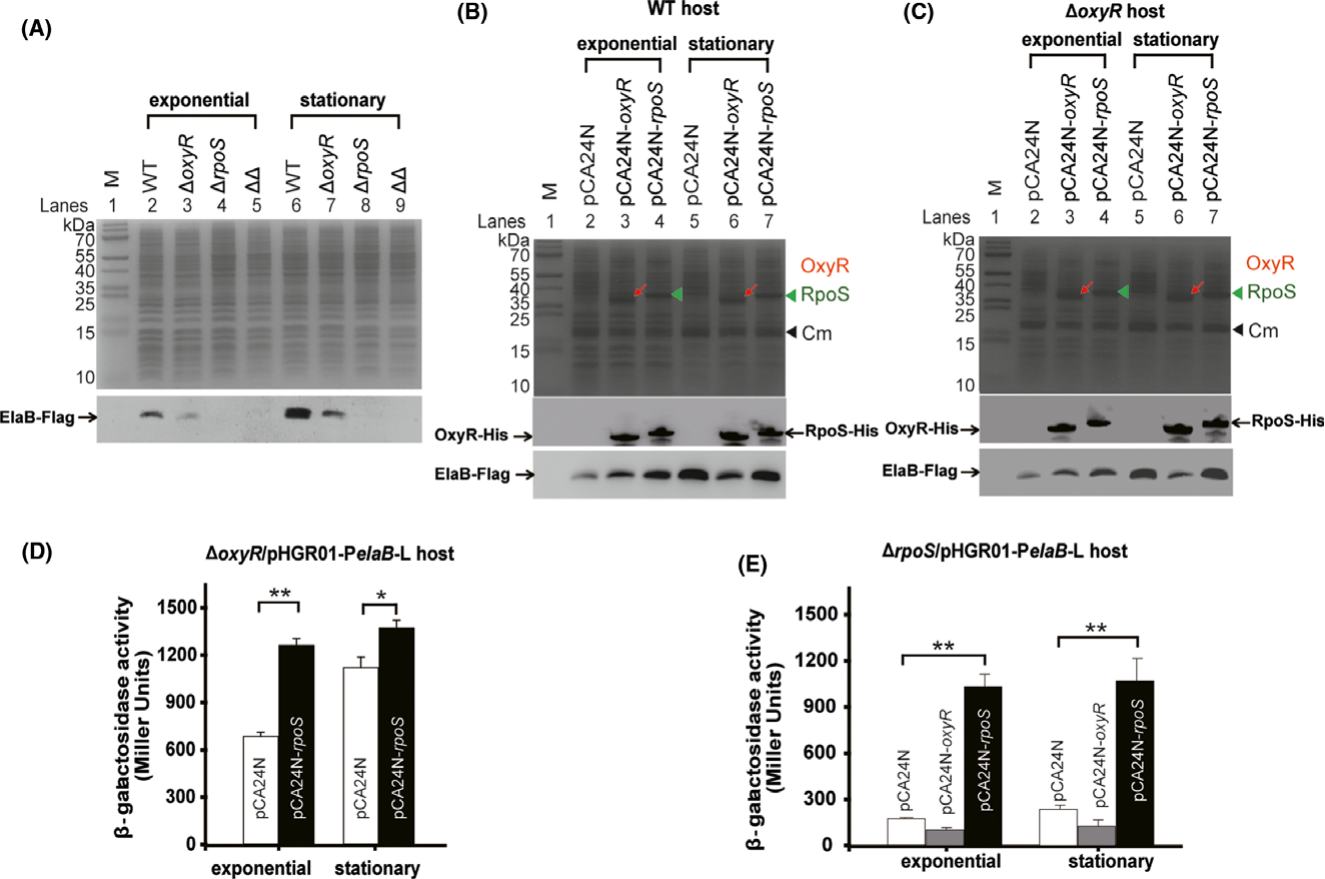
Promoter activity of *elaB* is regulated by OxyR in an RpoS‐dependent manner. (A) Production of ElaB‐Flag was determined using Western blotting for the BW25113 wild type (WT), Δ*oxyR*, Δ*rpoS* and ΔΔ cells. Same amount of total protein was loaded in each lane. The expression plasmids pCA24N‐*oxyR* and pCA24N‐*rpoS* were transferred into WT (B) and Δ*oxyR* (C) cells. Production of OxyR‐His (red arrows) and RpoS‐His (green triangles) was induced by 0.5 mM IPTG at OD_600_ ~ 0.1 for 2 h and 6 h. Cm indicates the chloramphenicol resistance protein. The levels of OxyR‐His, RpoS‐His and ElaB‐Flag were determined using Western blotting. Same amount of total protein was loaded in each lane. (D) Δ*oxyR*/pHGR01‐P*elaB*‐L cells expressing RpoS were induced with 0.5 mM IPTG for exponential phase and stationary phase, and β‐galactosidase activities were tested. The pCA24N vector was used as a negative control. (E) The Δ*rpoS*/pHGR01‐P*elaB*‐L cells expressing *oxyR* and *rpoS* were induced, and β‐galactosidase activities were determined as described in D. For statistical analysis, *P *<* *0.01 is marked as **.

Oxidative stress‐related genes including *dps* (Altuvia *et al*., 1994), *gor* (Becker‐Hapak and Eisenstark, 1995) and *hpI* (Ivanova *et al*., 1994) are regulated by both OxyR and RpoS, and we have shown that *elaB* is regulated by RpoS (Guo *et al*., 2017). Here, we found that *elaB* expression is also regulated by both OxyR and RpoS. To confirm this at protein level, we fused a 2× Flag tag to ElaB in the WT, ∆*oxyR*, ∆*rpoS* and ∆∆ cells (*rpoS* and *oxyR* double mutant). As observed by Western blotting, more ElaB was produced during the stationary phase compared to the exponential growth phase in wild‐type cells (Fig. 4A, lane 6 vs lane 2) and this could also be detected in the *oxyR* mutant cells (Fig. 4A, lane 7 vs 3). More importantly, less ElaB was produced in ∆*oxyR* cells compared to wild‐type cells during the exponential growth phase (Fig. 4A, lane 3 vs lane 2) and the stationary phase (Fig. 4A, lane 7 vs lane 6). As expected, ElaB was not produced in the ∆*rpoS* strain and in the ∆∆ strain (Fig. 4A, lanes 4–5, 8–9). To explore how OxyR and RpoS regulate *elaB* expression, we first overexpressed RpoS via pCA24N‐*rpoS* in the wild‐type and Δ*oxyR* strains. Results showed that ElaB production was induced by RpoS at the exponential growth phase but not the stationary phase (Fig. 4B and C). To further confirm this, we produced RpoS in the Δ*oxyR*/pHGR01‐P*elaB*‐L reporter strain and found that the promoter activity was induced by RpoS at a higher level at the stationary phase compared to the exponential growth phase (Fig. 4D). Thus, *elaB* expression when *rpoS* is overproduced is independent of OxyR. Next, we explored whether the regulation of OxyR on *elaB* is independent of RpoS using the ∆*rpoS*/pHGR01‐P*elaB*‐L reporter strain. As shown in Fig. 4E, OxyR was not able to induce the promoter activity in the absence of *rpoS*, indicating that the regulation of *elaB* by OxyR depends on RpoS. In addition, when OxyR or RpoS was overproduced in ∆∆ harbouring pHGR01‐P*elaB*‐L, as expected, RpoS but not OxyR induced the promoter activity of *elaB* (Fig. S4). Similarly, complementation of *rpoS* via pCA24N‐*rpoS* in the ∆*rpoS* and ∆∆ strains restored the production of ElaB protein, but complementation of *oxyR* via pCA24N‐*oxyR* was unable to do that (Fig. S5). Taken together, these results demonstrate that OxyR induces the expression of *elaB* in the exponential growth phase and that the regulation of OxyR is RpoS‐dependent.

Furthermore, using the ∆∆ strain, we found that expression of *elaB* was significantly higher in the stationary phase compared to the exponential growth phase even in the absence of both *oxyR* and *rpoS* (Fig. S4). This result implies that other forms of RNA polymerase might be involved with the expression of *elaB* during the stationary phase and that this form of regulation should be repressed by OxyR. Consistent with this idea, less ElaB was produced when OxyR was overproduced via pCA24N‐*oxyR* when compared to the empty plasmid in the stationary phase (Fig.  4B and C, lane 6 vs lane 5). Since the OxyR binding site overlaps the −35 and −10 regions, it is possible that OxyR acts to stimulate RpoS‐dependent transcription of *elaB* and also acts to repress transcription of *elaB* by some other form of RNA polymerase when needed.

### ElaB increases fitness in mixed populations

**Figure 5:**
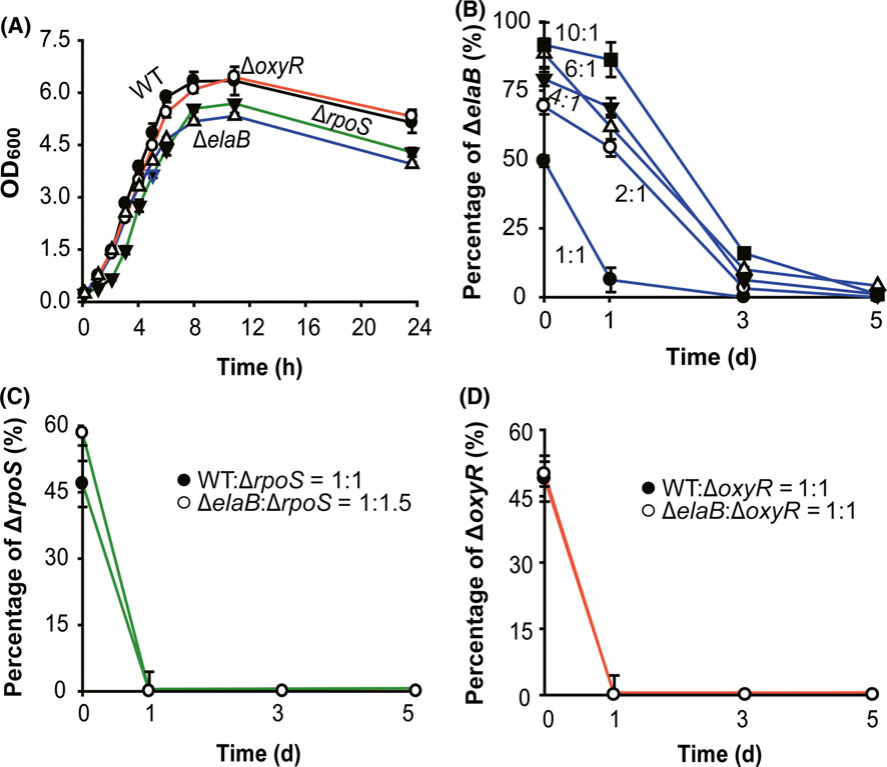
ElaB increases cell fitness. A. Growth of BW25113 wild type (WT), Δ*elaB*, Δ*oxyR* and Δ*rpoS*. B. Overnight cultures of WT and Δ*elaB*:*:km* were diluted to OD_600_ 0.1 and were cultured till OD_600_ 1.0. Then, different ratios of Δ*elaB*:*:km* and WT were mixed, and the percentages of Δ*elaB*:*:km* in total cells were determined at different time points. C. The Δ*rpoS*::*km* cells were mixed with WT and Δ*elaB* at the ratio of 1:1, and the percentages of Δ*rpoS*::*km* in total cells were determined at different time points. D. The Δ*rpoS*::*km* cells in (C) were replaced by Δ*oxyR*::*km,* and the percentages of Δ*oxyR*::*km* in total cells were determined at different time points.

Our previous study indicated that ElaB helps cells withstand oxidative stress and heat‐shock stress, indicating that ElaB may increase the fitness of cells. In the current study, we found that the growth of the ∆*elaB* strain was slower than the wild type in the stationary phase but not in the exponential phase (Fig. 5A). Next, we mixed exponential cultures of the ∆*elaB*::*km* and wild‐type strains at a cell ratio of 1:1, and the percentage of ∆*elaB*::*km* cells in the mixed population was determined using drop assays on LB plates supplemented with and without kanamycin. The percentage of ∆*elaB*::*km* cells in the mixed population was greatly reduced after 1 day, and a complete depletion of ∆*elaB*::*km* cells was observed after 3 days (Fig. 5B). Similar results were obtained when ∆*elaB*::*km* cells and wild‐type cells were inoculated at ratios of 2, 4, 6 and 10 (Fig. 5B). To exclude the possibility of killing effects of the wild‐type cells towards ∆*elaB*::*km* cells*,* we inoculated the wild‐type and ∆*elaB*::*km* cells with the filtered supernatant collected from the 5 day culture of wild‐type cells and no killing was observed for the two strains (data not shown). Additionally, we found that deletion of *rpoS* but not *oxyR* also reduced growth during the stationary phase (Fig. 5A). As expected, the ∆*rpoS*::*km* cells were less competitive than the wild‐type and the ∆*elaB* cells when co‐cultured by shaking (Fig. 5C). Similar results were also obtained when the ∆*oxyR*::*km* cells were used to co‐culture with wild‐type or the ∆*elaB* cells (Fig. 5D). To exclude the possible effect of the kanamycin resistance marker on the competition result, we further confirmed these results by qPCR using strains without this antibiotic marker gene (Fig. S6). We also conducted the competition study under microaerobic and oxidative stress conditions, and similar results were obtained (Fig. S7). Therefore, ElaB increases the cell fitness in a mixed population and the decreased fitness in *oxyR* and *rpoS* deletion mutants could be partially explained by the reduction in ElaB.

## Discussion

Recently, we demonstrated that the C‐tail anchored inner membrane protein ElaB protects cells against oxidative stress and heat shock, that it reduces persistence, and that the expression of *elaB* is regulated by RpoS (Guo *et al*., 2017). In this follow‐up study, we found that the expression of *elaB* is induced by oxidative stress by the transcriptional activator of the oxidative stress response, OxyR. OxyR binds to the promoter region of ElaB in the exponential growth phase, and the transcriptional regulation of OxyR to *elaB* is RpoS‐dependent. We further demonstrated that excess of OxyR inhibits ElaB production during stationary growth when RpoS is the master regulator. The mechanisms that bacteria use to defend against oxidative stress can be classified as either repairing damaged cellular components or enzymes involved in removing active oxygen species. C‐anchored inner membrane protein ElaB does not contain an enzymatic domain, and here, we show that ElaB protects cells against oxidative stress by maintaining cell membrane integrity.

Most proteins involved in oxidative stress are regulated by one or more regulators, including OxyR, RpoS or SoxRS (Farr and Kogoma, 1991). The regulation of the first two regulators is well‐characterized and occurs due to binding to specific regions in the promoter in *E. coli*. Here, we provide evidence that OxyR also induces the expression of *elaB* by binding to its promoter region. Similarly, OxyR activates the expression of *ahpC* and the divergently transcribed *dsbG* via two OxyR binding sites located at the intergenic region between *dsbG* and *ahpC* (Zheng *et al*., 2001a,b). Transcription of *ychF* which encodes a KatG inhibitor protein is repressed by OxyR, and this regulation activates the *katG* by decreasing the ATPase activity of YchF (Wenk *et al*., 2012). However, OxyR also acts as a repressor of antioxidant genes in bacteria (Zheng *et al*., 2001a,b; Teramoto *et al*., 2013); for example, *uxuA*,* uxuB*,* ygaQ*,* gntP* and b2653 are all possible OxyR‐repressed genes which are induced by one mM H_2_O_2_ and have OxyR binding sites in their promoter regions (Zheng *et al*., 2001a,b). In addition, we recently showed that *elaB* is also induced by RpoS (Guo *et al*., 2017), the stationary phase master regulator in *E. coli*. Here, we found that no ElaB was produced in the *rpoS* mutant strain during both the exponential and stationary phases, and OxyR no longer positively regulates *elaB* in the absence of RpoS. These results suggest that the regulation of *elaB* by OxyR is RpoS‐dependent. Other genes that participate in antioxidant activities are also regulated by both OxyR and RpoS (e.g. *gorA* and *dpS*) (Storz and Imlay, 1999). For example, the expression of a stationary phase‐induced gene *dps* is regulated by OxyR in actively growing cells but is regulated by RpoS during the stationary phase (Altuvia *et al*., 1994; Martinez and Kolter, 1997). In addition, expression of *oxyR* is positively regulated by the cAMP‐activated Crp protein during exponential growth and negatively regulated by RpoS when cells enter the stationary phase (GonzalezFlecha and Demple, 1997). We also found that the function of ElaB is not related to O_2_ concentrations (Figs S7 and S8) and that ElaB increases fitness in mixed populations. It remains to be determined whether ElaB participates in the removal of reactive oxygen species.

The oxidative stress response is involved in apoptosis and pathogenesis, and it usually overlaps with other stress responses including those related to antibiotic stress, heat shock, cold shock and starvation in bacteria (Farr and Kogoma, 1991; Battesti *et al*., 2011; Dale *et al*., 2015; Jara *et al*., 2015; Spaniol *et al*., 2015; Guo *et al*., 2017). Putative binding sites of some other regulators including RpoD and RpoH are also found in the promoter region of *elaB*, suggesting that ElaB might be involved in other stress responses. To date, three C‐tail anchored inner membrane proteins, YqjD, YgaM and ElaB, have been identified in *E. coli* (Yoshida *et al*., 2012). Expression of these three genes is all induced when cells enter the stationary phase (Yoshida *et al*., 2012); moreover, YqjD and ElaB are not produced in the absence of RpoS. YqjD binds to ribosomes at the N‐terminal region and may cause a functional defect in the translational activity of ribosomes (Yoshida *et al*., 2012). However, unlike YqjD, ElaB does not inhibit cell growth (Yoshida *et al*., 2012; Guo *et al*., 2017), suggesting these proteins may function differently. Thus, future studies are needed to elucidate the physiological functions of these C‐tail anchored inner membrane proteins in bacteria.

## Experimental procedures

### Bacterial strains, plasmids and growth conditions

The *E. coli* strains and plasmids used in this study are listed in Table 1. Luria‐Bertani (LB) medium was used in all the experiments. The Keio collection (Baba *et al*., 2006) and the ASKA library (Kitagawa *et al*., 2005) were used for deleting and overexpressing single gene. Chloramphenicol (30 μg ml^−1^) was used for maintaining pCA24N‐based plasmids, and kanamycin (50 μg ml^−1^) was used for maintaining the pET28b‐*oxyR* plasmid.

**Table 1:** Bacterial strains and plasmids used in this study

Bacterial strains/plasmids	Description	Source
*E. coli* K12 BW25113 strains		
Wild‐type	*lacI* ^q^ *rrnB*_T14_ Δ*lacZ*_WJ16_ *hsdR*514 Δ*araBAD*_AH33_ Δ*rhaBAD*_LD78_ *rph*‐*1*	Baba *et al*. (2006)
Δ*elaB*::*km*	Δ*elaB* km^R^	Baba *et al*. (2006)
Δ*elaB*	Δ*elaB* Δkm^R^	This study
Δ*rpoS*::*km*	Δ*rpoS* km^R^	Baba *et al*. (2006)
Δ*rpoS*	Δ*rpoS* Δkm^R^	This study
Δ*oxyR*::*km*	Δ*oxyR* km^R^	Baba *et al*. (2006)
Δ*oxyR*	Δ*oxyR* Δkm^R^	This study
Δ*rpos*Δ*oxyR*	Δ*rpoS* Δ*oxyR* Δkm^R^	This study
*elaB*::2×Flag	Two Flag sequences inserted before the *elaB* stop codon in the wild‐type strain	This study
*elaA*::2×Flag	Two Flag sequences inserted before the *elaA* stop codon in the wild‐type strain	This study
*oxyR*::2×Flag	Two Flag sequences inserted before the *oxyR* stop codon in the wild‐type strain	This study
Δ*rpoS elaB*::2×Flag	Two Flag sequences inserted before the *elaB* stop codon in the Δ*rpoS* strain	This study
Δ*oxyR elaB*::2×Flag	Two Flag sequences inserted before the *elaB* stop codon in the Δ*oxyR* strain	This study
Δ*rpoS*Δ*oxyR elaB*::2×Flag	Two Flag sequences inserted before the *elaB* stop codon in the Δ*rpoS* Δ*oxyR* strain	This study
Plasmids		
pCA24N	Cm^R^; *lacI* ^q^	Kitagawa *et al*. (2005)
pCA24N‐*elaB*	Cm^R^; *lacI* ^q^, P_*T5‐lac*_::*elaB*	Kitagawa *et al*. (2005)
pCA24N‐oxyR	Cm^R^; *lacI* ^q^, P_*T5‐lac*_::*rpoE*	Kitagawa *et al*. (2005)
pCA24N‐*rpoS*	Cm^R^; *lacI* ^q^, P_*T5‐lac*_::*rpoS*	Kitagawa *et al*. (2005)
pET28b	Km^R^, *lacI* ^q^, P_T7_ expression vector	Novagen
pET28b‐*oxyR*	Km^R^, *lacI* ^q^, pET28b P_*T7‐lac*_:: *oxyR* with *oxyR* C‐terminus His‐tagged	This study
pMD19	Amp^R^; promoterless T simple vector	Takara
pMD19‐*elaB‐flag*	300 bp promoter to elaB stop codon in *elaB*::2×Flag was cloned into pMD19 vector	This study
pCP20	Amp^R^ and Cm^R^; temperature‐sensitive replication, thermal induction of FLP recombinase synthesis	Datsenko and Wanner (2000)
pKD46	Amp^R^, λ Red recombinase expression	Datsenko and Wanner (2000)
pHGR01	Km^r^, R6K ori, promoterless‐*lacZ* reporter vector	Guo *et al*. (2017)
pHGR01‐P*elaB*‐L	Fused *elaB* promoter containing the OxyR binding site with *lacZ* in pHGR01	This study
pHGR01‐P*elaB*‐S	Fused *elaB* promoter in the absence of OxyR binding site with *lacZ* in pHGR01	Guo *et al*. (2017)
pHGR01‐P*elaB*‐SM	OxyR binding site was mutated in pHGR01‐P*elaB*‐S	This study

Cm^R^ and Km^R^ indicate chloramphenicol and kanamycin resistance respectively.

### qRT‐PCR

Total RNA was isolated using an RNA isolation kit (Invitrogen, Carlsbad, CA). DNase was applied during the RNA isolation process to avoid contamination by DNA. A total of 50 ng of total RNA was used for qRT‐PCR using the *Power* SYBR^®^ Green RNA‐to‐C_T_™ *1‐Step* Kit and the StepOne™ Real‐Time PCR System (Applied Biosystems, Foster City, CA, USA). All the genes were normalized to *rrsG*. Fold changes for induction or repression of *elaB* under different conditions were calculated using the formula described previously (Guo *et al*., 2014).

### Construction of 2× Flag fused strains of chromosomally encoded *elaA*,* elaB* and *oxyR*

To construct chromosomal copies of *elaB*::2× Flag with the native promoter, the one step inactivation method (Datsenko and Wanner, 2000) was applied to fuse 2× Flag before the stop codon of *elaB* to generate protein ElaB‐Flag. The kanamycin resistance (Km^r^) gene, which is bordered by FLP recombination target (FRT) sites, was amplified from plasmid pKD4 using primers *elaB*‐KM‐f and *elaB‐*KM‐r. The PCR product is a DNA fragment carrying 2× Flag and the Km^r^ cassette flanked by about 60 nt regions up‐ and downstream of the *elaB* stop codon. The PCR products were purified using a gel extraction kit (Qiagen, Valencia, CA, USA), and the purified fragments were electroporated into BW25113/pKD46 competent cells. Strain of BW25113 *elaB*::2× Flag was confirmed by PCR followed by DNA sequencing using primers of *elaB*‐conf‐f and *elaB*‐conf‐r. The same procedures to construct *elaB*::2× Fag strain were performed to fuse 2× Flag before the stop codon of the *elaA* and *oxyR* genes.

### Generation of the double‐mutant strain

The double‐gene knockout mutant of *oxyR* and *rpoS* (ΔΔ) was constructed using P1 transduction based on the single deletion mutants available in the Keio collection (Baba *et al*., 2006; Williams, 2011). P1 transduction was first performed to transfer the Δ*rpoS*::*km* mutation to the Δ*oxyR* mutant to obtain strain ΔΔ::*km*. Similarly, the strain with the fused 2× Flag before the stop codon of *elaB* (BW25113 *elaB*::2× Flag) was used as the donor for P1 transduction. The correct constructions were confirmed with the primers listed in Table S1. The kanamycin resistance cassette from the newly constructed double‐mutant strain was removed with the helper plasmid pCP20 (Datsenko and Wanner, 2000).

### Microscopy

To evaluate cell membrane integrity, the Live/Dead *Bac*Light™ Bacterial Viability Kit (Molecular Probes, Eugene, OR, USA) was used. Overnight cultures were diluted and cultured to a turbidity at 600 nm of 1.0, and cells were harvested by centrifugation (3500 × *g*, 2 min), washed and re‐suspended in 0.85% NaCl. Cells were then treated with H_2_O_2_ for 10 min followed by staining with 0.15 mM propidium iodine and 0.025 mM SYTO 9 dye for 15 min at the ambient temperature. Bacterial cells were imaged using a Zeiss Axiovert fluorescence microscope (Carl Zeiss Inc., Thornwood, NY, USA). The same cells were also used for the plasma membrane‐specific dye red‐fluorescent FM^®^ 4‐64 (Thermo Fisher Scientific, Rockford, IL) and observed under the same conditions. For the lipid peroxidation staining, stationary cells were collected, washed and stained with the fluorescent radio‐probe dye C11‐BODIPY (Thermo Fisher Scientific) for indexing lipid peroxidation and antioxidant efficacy in model membrane systems (Drummen *et al*., 2002), as mentioned above.

### Tricine‐SDS‐PAGE and Western blotting analysis

Tricine‐SDS‐PAGE and Western blotting were performed to determine production levels of the ElaB‐Flag, ElaA‐Flag and OxyR‐Flag. For cells treated with H_2_O_2_, BW25113 *elaB*::2× Flag, *elaA*:: 2× Flag and *oxyR*:: 2× Flag were cultured to a turbidity of 1.0 at 600 nm and treated with 5 mM H_2_O_2_ for 15 min, 30 min and 60 min. Cells containing pCA24N, pCA24N‐*oxyR* and pCA24N‐*rpoS* were diluted to a turbidity of 0.1 in LB with 30 μg ml^−1^ chloramphenicol, then 0.5 mM IPTG was added to induce *rpoS* and *oxyR* expression for 2 and 6 h, and cells were washed with TE buffer. Samples were sonicated, and the protein concentration was measured by using a Bi Yuntian BCA assay kit (Haimen, China). Protein was denatured at 95°C for 5 min. A total of 25 μg total protein for each sample was loaded for Tricine‐SDS‐PAGE, and 2.5 μg of total protein was loaded for the Western blotting with primary antibodies raised against the Flag tag (for ElaB, ElaA and OxyR produced by chromosome) or His tag (for RpoS and OxyR produced via plasmids) (Cell Signaling Technology, Danvers, MA, USA), and horseradish peroxidase‐conjugated goat anti‐mouse was used as the secondary antibody (Bio‐Rad, Richmond, CA, USA).

### 5′ rapid amplification of cDNA ends (RACE)

Total RNA was isolated as mentioned above for qRT‐PCR. The following procedures were conducted using SMARTer@RACE 5′ kit (Takara, Japan) according to the manufacturer's protocol.

### Electrophoretic mobility shift assay (EMSA)

EMSAs were conducted as described (Lee and Gralla, 2001; Zhao *et al*., 2005). Briefly, DNA fragments were amplified using the primer pairs shown in Table S1. PCR amplicons were gel purified with a QIAquick Gel Extraction Kit (Qiagen), and the purified products were labelled with the Pierce™ biotin 3′ end DNA labelling kit (Thermo Fisher Scientific, Rockford, IL). The binding reaction was performed with the non‐specific competitor DNA (poly dI‐dC) and NP‐40 in buffer containing 10 mM HEPES (pH 7.3), 20 mM KCl, 1 mM MgCl_2_ and 5% glycerol at 25°C for 2 h. The final mixtures were run on a 6% DNA retardation gel (Invitrogen), transferred to a nylon membrane and UV cross‐linked. Chemiluminescence was performed with the LightShift Chemiluminescent EMSA Kit (Thermo Fisher Scientific) according to the manufacturer's protocol.

### β‐galactosidase activity assay

The reporter plasmids pHGR01‐P*elaB*‐L and pHGR01‐P*elaB*‐SM were constructed following previous procedures (Guo *et al*., 2017) with primers shown in Table S1. BW25113 wild‐type and Δ*oxyR* strains harbouring either of the two plasmids or pHGR01‐P*elaB*‐S (Guo *et al*., 2017) were cultured to a turbidity at 600 nm of 1.0, and 800 μl cultures were diluted with 4 ml PM2 buffer. The reaction was conducted, the absorbance was measured at 420 nm, and then, the β‐galactosidase activity (Miller units) was calculated as previously described (Karimova *et al*., 2005; Frias and Flores, 2015). For the *rpoS* and *oxyR* overexpression experiments, cells carrying pCA24N‐*rpoS* and pCA24N‐*oxyR* were cultured to a turbidity at 600 nm 0.1, 0.5 mM IPTG was added to induce protein expression for 2 h and 6 h, and β‐galactosidase activity was measured.

### Competition assay

Overnight cultures were diluted to a turbidity at 600 nm of 0.1 in LB medium and incubated at 37°C with 250 RPM shaking until cultures reached a turbidity of 0.8–1.0. Then, the same number of cells of the two strains for competition were mixed and cultured under different conditions for 1 day. The two conditions are standard growth with LB medium and growth in a BACTROX‐2 microaerobic chamber (SHEL LAB, USA) equilibrated to a 5% O_2_ and 10% CO_2_ atmosphere condition. The cells were diluted 100‐fold and recultured every day for 5 days. The cells of each day were dropped on LB with and without kanamycin plates and cultured overnight, and colonies were counted, and then, the ratios of cells were calculated. To exclude the possible effect of kanamycin resistance marker on the competition result, we removed the marker gene with pCP20 and performed the competition assay under the same conditions. After grew on LB plates, 96 colonies were randomly selected for each time point and amplified by qPCR with primers flanking *elaB* gene.

### Statistical analysis

Data are presented as means ± *SE* of three or more independent cultures. Statistical significance was assessed using two‐tailed unpaired Student's *t*‐test.

## Conflict of interests

The authors declare no competing financial interests.

## Supporting information


**Table S1.** Oligonucleotides used for cloning, qRT‐PCR, flag insertion via the chromosomal copy of *elaB,* and probe amplification.
**Fig. S1.** Production of the ElaB‐Flag fused protein complemented the oxidative stress sensitivity of the *elaB* mutant strain.
**Fig. S2.** ElaB mutation weakens cell membrane.
**Fig. S3.** ElaB protects the cell membrane against exogenously added H2O2.
**Fig. S4.** OxyR and RpoS were expressed in the *oxyR* and *rpoS* double mutant (ΔΔ) ΔΔ/pHGR01‐PelaB‐L, and β‐galactosidase activities were determined as in Fig. 4D.
**Fig. S5.** The expression plasmids pCA24N‐*oxyR* and pCA24N‐*rpoS* were transferred into the Δ*rpoS* and ΔΔ cells.
**Fig. S6**. Competition of WT, Δ*elaB*, Δ*oxyR* and Δ*rpoS* was tested, and all the three mutant strains without kanamycin (Km) resistance.
**Fig. S7.** ElaB increases cell fitness under microaerobic and oxidative stress conditions.
**Fig. S8.** Growth of BW25113 wild type and Δ*elaB* strains under microaerobic condition.
