# Supplementary material for: Resistance to oxidative stress by inner membrane protein ElaB is regulated by OxyR and RpoS
Source: Microb Biotechnol. 2019 Jan 17;12(2):392–404. doi: 10.1111/1751-7915.13369 (PMC6389858; doi:10.1111/1751-7915.13369)

# Supplemental Material

# Resistance to Oxidative Stress by Inner Membrane Protein ElaB is Regulated by OxyR and RpoS

Yunxue Guo[1#], Yangmei Li[1,2], Waner Zhan[1,2], Thomas K. Wood[3, 4], and Xiaoxue Wang[1,2]

[1]CAS Key Laboratory of Tropical Marine Bio-resources and Ecology, Guangdong Key Laboratory of Marine Materia Medica, RNAM Center for Marine Microbiology, South China Sea Institute of Oceanology, Chinese Academy of Sciences, Guangzhou 510301, China

[2]University of Chinese Academy of Sciences, Beijing 100049, China

[3]Department of Chemical Engineering, [4]Department of Biochemistry and Molecular Biology, Pennsylvania State University, University Park, PA 16802-4400, USA

# To whom correspondence should be addressed. Tel: +86 20 89267515; Fax: +86 20 89235490; E-mail: yunxueguo@scsio.ac.cn

**Running title:** ElaB is regulated by OxyR and RpoS

**Keywords:** Oxidative stress, inner membrane protein, membrane integrity

**Table S1.** Oligonucleotides used for cloning, qRT-PCR, flag insertion via the chromosomal copy of *elaB,* and probe amplification. f indicates the forward primer and r indicates the reverse primer. KM indicates kanamycin resistance gene.

| Purpose/Name | Sequence (5'-3') |
|---|---|
| **qRT-PCR** | |
| *elaB*-f | ACGACCTGACGCTGCTTAGT |
| *elaB*-r | AGCACGATAAACTGCCTGCT |
| *oxyR*-f | TGAGGTGAAAGTCCTTAAAGAGATG |
| *oxyR*-r | GTCTGTGCTTCATGCAGATACATT |
| *elaA*-f | CTTTATGCCTTATTACAACTGCG |
| *elaA*-r | CACTGACAATCACCCGACCT |
| *rpoS*-f | TAACTTGCGTCTGGTGGTAAAA |
| *rpoS*-r | TGTGAATCGGCAAACGAATAGT |
| *rrsG*-f | TATTGCACAATGGGCGCAAG |
| *rrsG*-r | ACTTAACAAACCGCCTGCGT |
| **Flag insertion in chromosome** | |
| *elaA* -KM-f | GAAGATGGTATCCCACACATTGGGATGGCACGCGAGGTAATTCAGGCGGATTACAAGGAT<br>GACGACGATAAGGATTACAAGGATGACGACGATAAGTGAGTGTAGGCTGGAGCTGCTTC |
| *elaA*-KM-r | GCGTAAAACCTGTCACTAACTATAGACAAGGGTTGTTGATCCATATGAATATCCTCCTT |
| *elaA*-conf-f | ATAATCGCCATATTCTCGG |
| *elaA*-conf-r | CACTAAGCAGCGTCAGGTC |
| *elaB*-KM-f | GTGGGCGCGGCCGTTGGGCTGGTACTAGGACTGTTGCTGGCACGCCGTGATTACAAGGAT<br>GACGACGATAAGGATTACAAGGATGACGACGATAAGTGAGTGTAGGCTGGAGCTGCTTC |
| *elaB*-KM-r | TACTACAAAAAAAATGCAGTACCCCGGTGTAGGGAGGTTTCCATATGAATATCCTCCTT |
| *elaB*-conf-f | GGCGAAGCAGGCAGTTTATC |
| *elaB*-conf-r | GGAATCTCTTGCGACAAATGG |
| *oxyR*-KM-f | ATGGATGGCCATTTCGATAAAGTTTTAAAACAGGCGGTTGATTACAAGGATGACGACGA |
| *oxyR*-KM-r | ACGATGGCGGAAGCCTATCGGGTAGCTGCGTTAAACGGTTCCATATGAATATCCTCCTT |
| *oxyR*-conf-f | TGCATGAAGCACAGACCCAC |
| *oxyR*-conf-r | CCATCCACGCATTTACGACA |
| **Cloning** | |
| pHGR01-P*elaB*-L-f | CGGTGTTTGTTGTCGAGCAGA |
| pHGR01-P*elaB*-r | TCTCGTTCTCCATTTGCGTAAAACC |
| pHGR01-P*elaB*-SM-r2 | TGGACACACACAGTCCGACCTGAAGTTTACTCTCAAGAATCCCAATGTGTGGGATACCA |
| pHGR01-P*elaB*-SM-r3 | TCTCGTTCTCCATTTGCGTAAAACCTGTCACTAACTATAGATGGACACACACAGTCCGA |
| pHGR01-f | CGTCAATTATTACCTCCACG |
| pHGR01-r | GTGCTGCAAGGCGATTAAG |
| pET28b-*oxyR*-f | CTAGTCTAGAGTTTAACTTTAAGAAGGAGATATAATGAATATTCGTGATCTTGAGTACC |
| pET28b-*oxyR*-r | CCCAAGCTTTTAGTGATGATGATGATGATGAACCGCCTGTTTTAAAACTTTATCGAAAT |
| pET28b-f | TAATACGACTCACTATAGGG |
| pET28b-r | TATGCTAGTTATTGCTCAG |
| pMD19-*elaB-flag*-f | GAGCCGGTCGTTATAGGTCGGGTGATTGTCAGTGAAGCGTTGCGTGGCGAAAAAGT |
| pMD19-*elaB-flag*-r | TTACTTATCGTCGTCATCCTTGTAATCCTTATCGTCGTCATCCTTGTAATCACGGC |
| M13-f | TGTAAAACGACGGCCAGT |

| | |
|---|---|
| RV-M | AGCGGATAACAATTTCACACAGGA |
| **5' RACE** | |
| GSP-*elaB* | GATTACGCCAAGCTTGAGGAGCGGAGCACCTCTTCCAGTGTT |
| **Probe amplification** | |
| P*elaB*-S-f | ACTGGCCTGATAAGCCTGTTTACC |
| P*elaB*-S-r | TCTCGTTCTCCATTTGCGTAAAAC |
| P*elaB*-SM-r | TCTCGTTCTCCATTTGCGTAAAACCTGTCACTAACTCCCCACAAGGGTTGTTGATTACG |

**Figure S1. Production of the ElaB-Flag fused protein complemented the oxidative stress sensitivity of the *elaB* mutant strain.** Overnight cultures were diluted, cultured to a turbidity of 600 nm of 1.0, treated with 20 mM $H_2O_2$ for 10 minutes, and then cell survival (%) was assayed. Three independent cultures were used.

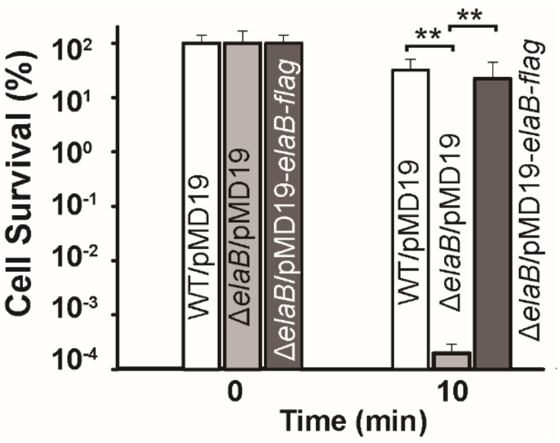

**Figure S2 ElaB mutation weakens cell membrane.** Cells were treated with $H_2O_2$ as indicated in Figure 1A and stained with plasma membrane specific red-fluorescent dye FM® 4-64. WT cells before (**A, B**) and after (**C, D**) $H_2O_2$ treatment were observed with a fluorescent microscope. The percentage of cells with weakened membranes are marked with white arrows. Three independent cultures for each strain were used and about 1000 cells in each culture were observed, and only one representative image for each strain was shown here. The percentages of cells with weak cell membrane were calculated.

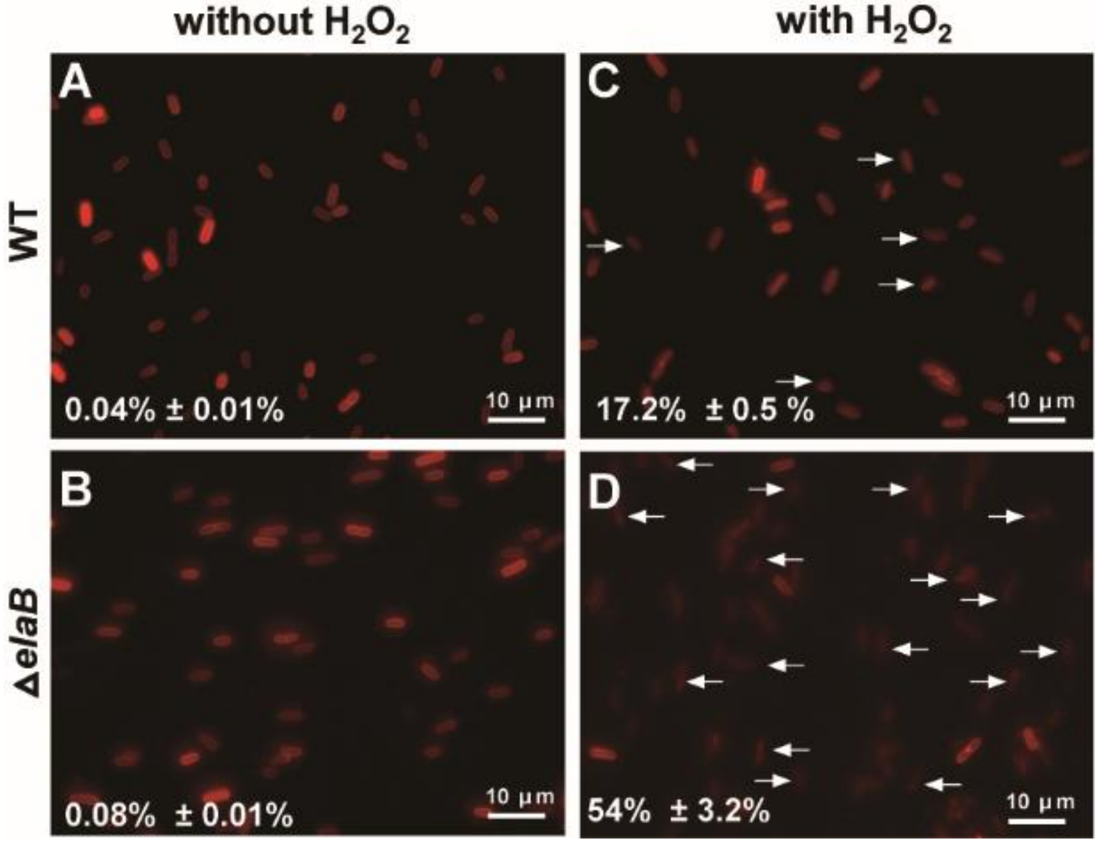

**Figure S3. ElaB protects the cell membrane against exogenously added $H_2O_2$**. TEM micrographs of wild-type cells before (**A**) and after (**C**) 50 mM $H_2O_2$ treatment for 30 min. The ultrastructure of the △*elaB* strain treated without (**B**) and with (**D**) 50 mM $H_2O_2$ for 30 min are also shown. Cells were cultured and collected as mentioned in the Material and Methods section. The ultrastructure of the cell membrane is indicated with a red arrow for a representative cell in each panel.

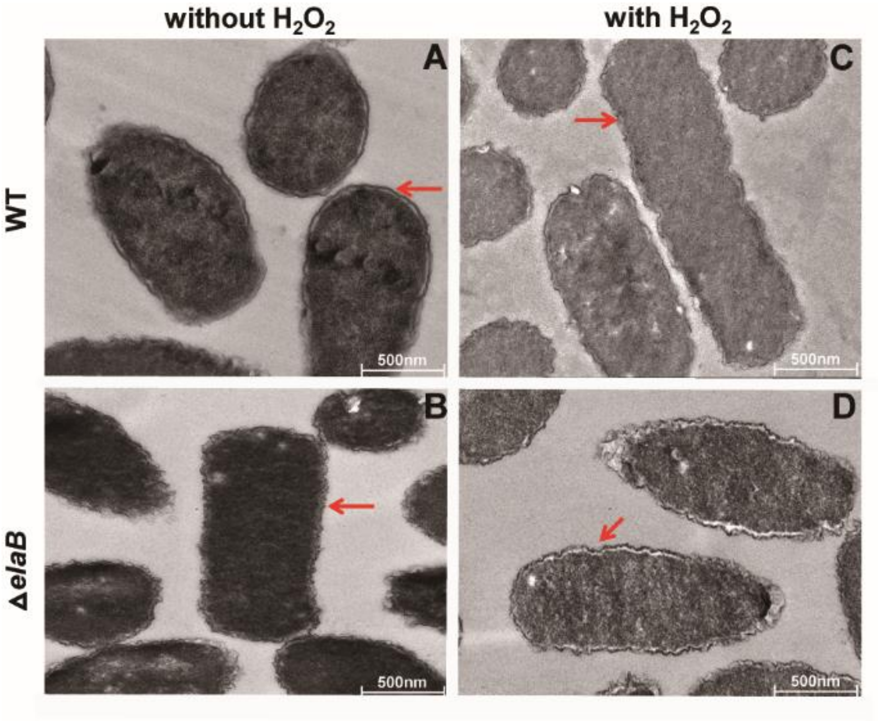

**Figure S4** OxyR and RpoS were expressed in the *oxyR* and *rpoS* double mutant (ΔΔ) ΔΔ/pHGR01-P*elaB*-L, and β-galactosidase activities were determined as in Figure 4D. Three independent cultures for each strain were used. For statistical analysis, *p*< 0.01 is marked as **.

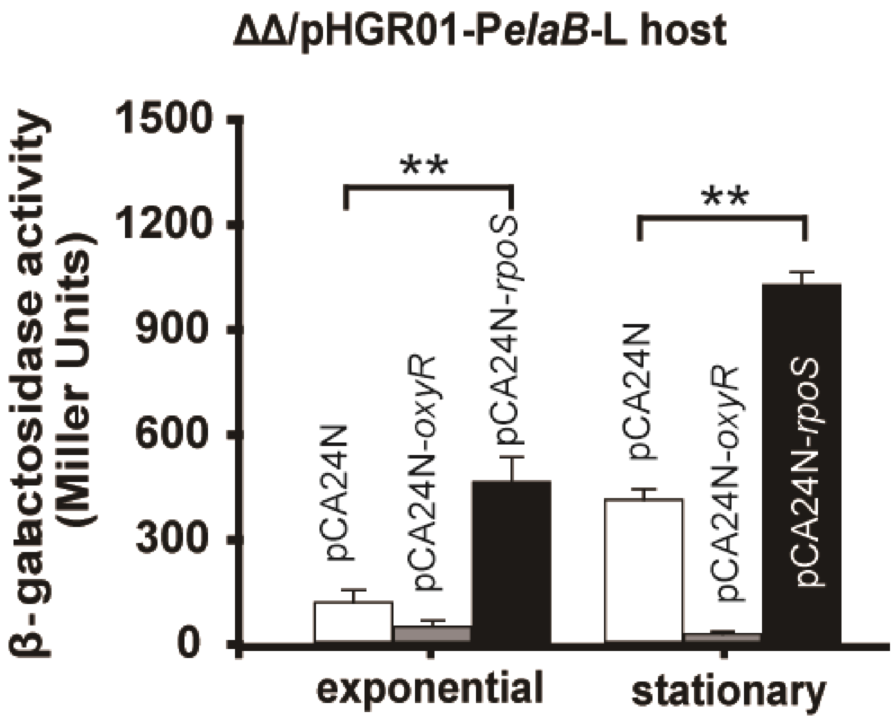

**Figure S5.** The expression plasmids pCA24N-*oxyR* and pCA24N-*rpoS* were transferred into the Δ*rpoS* and ΔΔ cells. Production of OxyR (red arrows) and RpoS (green triangles) was induced by 0.5 mM IPTG at OD600 0.1 for 2 h and 6 h. Cm indicates the chloramphenicol resistance protein. The levels of OxyR, RpoS and ElaB were determined in Δ*rpoS* (A) and ΔΔ (B). The same amount of total protein was loaded as controls.

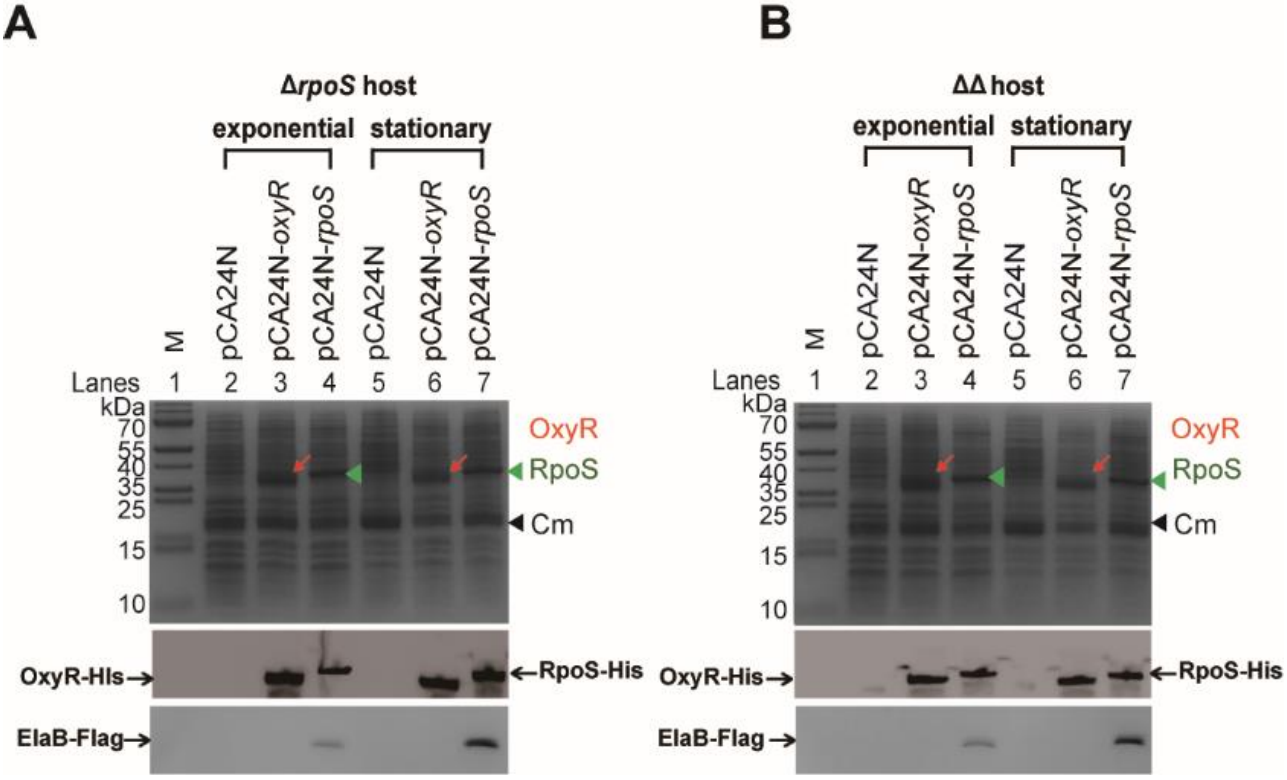

**Figure S6**. Competition of WT, Δ*elaB*, Δ*oxyR* and Δ*rpoS* was tested, and all the three mutant strains without kanamycin (Km) resistance. **(A)** Overnight cultures of WT and Δ*elaB* were diluted to $OD_{600}$ 0.1 and were cultured till $OD_{600}$ 1.0. Then different ratios of Δ*elaB* and WT were mixed, and the percentages of Δ*elaB* in total cells were determined at different time points by PCR amplification of 96 randomly selected colonies using primers flanking the *elaB* gene region. **(B)** The Δ*rpoS* cells were mixed with WT and Δ*elaB* at the ratio of 1:1, and the percentages of Δ*rpoS* in total cells were determined at different time points. **(C)** The Δ*rpoS* cells in **(B)** were replaced by Δ*oxyR* and the percentages of Δ*oxyR* in total cells were determined at different time points. Triplicates were used for each strain, and data were shown as mean± SD.

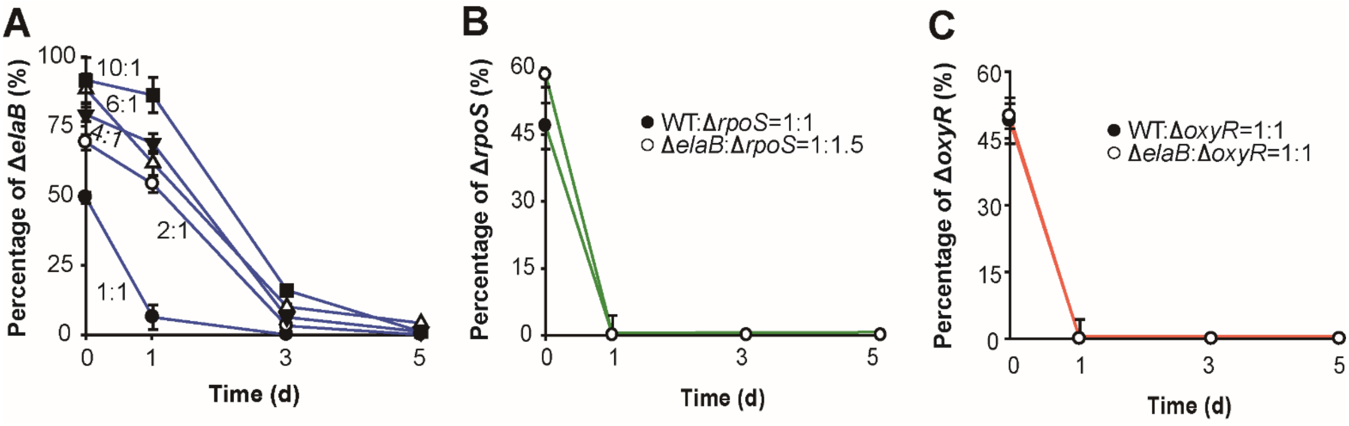

**Figure S7. ElaB increases cell fitness under microaerobic and oxidative stress conditions.** Overnight cultures of BW25113 wild type (WT) and Δ*elaB*::*km* were diluted to $OD_{600}$ 0.1 and were cultured till $OD_{600}$ 1.0. Then the same number of Δ*elaB*::*km* and WT cells were mixed and cultured under two conditions: (i) The mixed cells are treated with 1 mM $H_2O_2$ and the $H_2O_2$ was added again every day when the cells are re-cultured; (ii) The mixed cells are cultured in BACTROX-2 microaerobic chamber (SHELLAB, USA) equilibrated to a 5% $O_2$ and 10% $CO_2$ atmosphere. The percentages of Δ*elaB*::*km* in total cells were determined at different time points. Triplicates of each strain were used and error bar indicates standard error of mean (n = 3).

**A** **B**

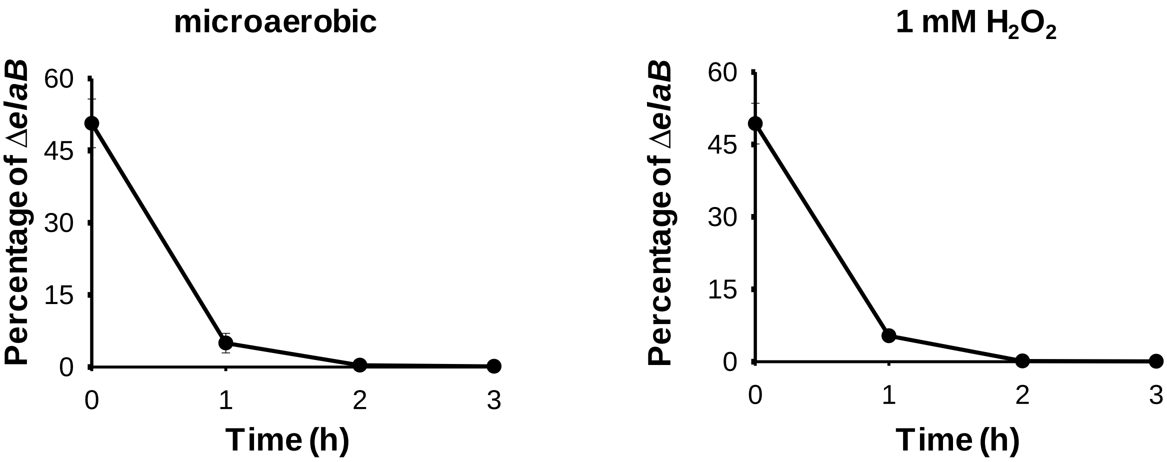

**Figure S8. Growth of BW25113 wild type and Δ*elaB* strains under microaerobic condition.** Cells were cultured in LB medium overnight. Then they were diluted to a turbidity at 600 nm 0.1, and cells were incubated in BACTROX-2 microaerobic chamber (SHELLAB, USA) equilibrated to a 5% $O_2$ and 10% $CO_2$ atmosphere, and turbidity (A) and CFU (B) were determined at indicated time points. Triplicates of each strain were used and error bar indicates standard error of mean (n = 3).

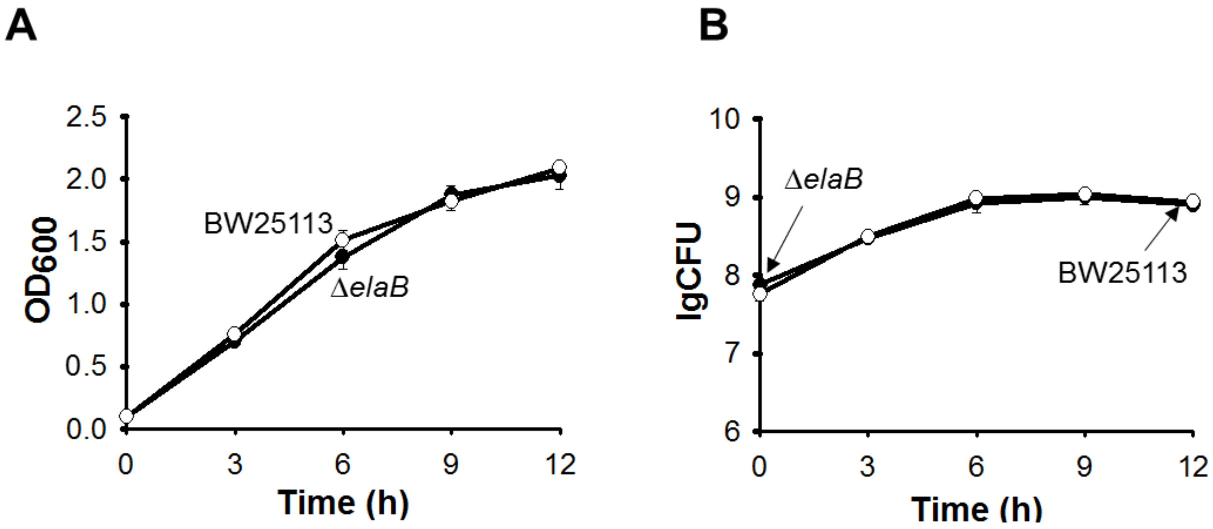

Supplement: Supplementary file 1 — Table S1. Oligonucleotides used for cloning, qRT‐PCR, flag insertion via the chromosomal copy of elaB, and probe amplification. Fig. S1. Production of the ElaB‐Flag fused protein complemented the oxidative stress sensitivity of the elaB mutant strain. Fig. S2. ElaB mutation weakens cell membrane. Fig. S3. ElaB protects the cell membrane against exogenously added H2O2. Fig. S4. OxyR and RpoS were expressed in the oxyR and rpoS double mutant (ΔΔ) ΔΔ/pHGR01‐PelaB‐L, and β‐galactosidase activities were determined as in Fig. 4D. Fig. S5. The expression plasmids pCA24N‐oxyR and pCA24N‐rpoS were transferred into the ΔrpoS and ΔΔ cells. Fig. S6. Competition of WT, ΔelaB, ΔoxyR and ΔrpoS was tested, and all the three mutant strains without kanamycin (Km) resistance. Fig. S7. ElaB increases cell fitness under microaerobic and oxidative stress conditions. Fig. S8. Growth of BW25113 wild type and ΔelaB strains under microaerobic condition. [file MBT2-12-392-s001.pdf]
